# Global and Phylogenetic Distribution of Quorum Sensing Signals, Acyl Homoserine Lactones, in the Family of Vibrionaceae

**DOI:** 10.3390/md12115527

**Published:** 2014-11-20

**Authors:** Bastian Barker Rasmussen, Kristian Fog Nielsen, Henrique Machado, Jette Melchiorsen, Lone Gram, Eva C. Sonnenschein

**Affiliations:** 1 Department of Systems Biology, Technical University of Denmark, Matematiktorvet bldg 301, DK-2800 Kgs, Lyngby, Denmark; E-Mails: bbara@bio.dtu.dk (B.B.R.); jeme@bio.dtu.dk (J.M.); gram@bio.dtu.dk (L.G.); 2 Department of Systems Biology, Technical University of Denmark, Søltofts Plads bldg 221, DK-2800 Kgs, Lyngby, Denmark; E-Mail: kfn@bio.dtu.dk; 3 Novo Nordisk Foundation Center for Biosustainability, Technical University of Denmark, Kogle Allè 6, DK-2970 Hørsholm, Denmark; E-Mail: henma@biosustain.dtu.dk

**Keywords:** quorum sensing, acyl homoserine lactones, Vibrionaceae, marine bacteria, diversity

## Abstract

Bacterial quorum sensing (QS) and the corresponding signals, acyl homoserine lactones (AHLs), were first described for a luminescent *Vibrio* species. Since then, detailed knowledge has been gained on the functional level of QS; however, the abundance of AHLs in the family of Vibrionaceae in the environment has remained unclear. Three hundred and one Vibrionaceae strains were collected on a global research cruise and the prevalence and profile of AHL signals in this global collection were determined. AHLs were detected in 32 of the 301 strains using *Agrobacterium tumefaciens* and *Chromobacterium violaceum* reporter strains. Ethyl acetate extracts of the cultures were analysed by ultra-high performance liquid chromatography-high resolution mass spectrometry (MS) with automated tandem MS confirmation for AHLs. *N*-(3-hydroxy-hexanoyl) (OH-C6) and *N*-(3-hydroxy-decanoyl) (OH-C10) homoserine lactones were the most common AHLs found in 17 and 12 strains, respectively. Several strains produced a diversity of different AHLs, including *N*-heptanoyl (C7) HL. AHL-producing Vibrionaceae were found in polar, temperate and tropical waters. The AHL profiles correlated with strain phylogeny based on gene sequence homology, however not with geographical location. In conclusion, a wide range of AHL signals are produced by a number of clades in the Vibrionaceae family and these results will allow future investigations of inter- and intra-species interactions within this cosmopolitan family of marine bacteria.

## 1. Introduction

Quorum sensing (QS) is a process induced by cell population density and allows bacteria to sense and act on their local environment, as well as, communicate both within and between species [1]. The essence underlying the mechanism of QS is based on the production and accumulation of signalling molecules called autoinducers. When the threshold concentration of the signals is reached, they bind to receptor proteins that then act as either transcriptional activators or repressors [2]. The QS signals are small molecules such as acyl homoserine lactones (AHLs) produced by Gram-negative bacteria, autoinducer-2 (AI-2) used by Gram-negative and Gram-positive bacteria and oligopeptides which are utilized by Gram-positive bacteria [1,3]. QS systems are involved in the regulation of several different bacterial phenotypes such as biofilm formation, bioluminescence, virulence and production of bioactive compounds [1,4,5,6].

Vibrionaceae is a large family of Gram-negative marine, facultative anaerobic bacteria belonging to the Gammaproteobacteria. This family includes several genera of which the largest are *Vibrio* and *Photobacterium* that include human and fish pathogens such as* Vibrio cholerae*,* V. anguillarum,* and *V. vulnificus*. Also included are the algal and squid symbionts such as *V. pomeroyi*, *V. aestuarianus*, and* Aliivibrio fischeri* (formerly known as* Vibrio fischeri*) [6,7,8,9]. Quorum sensing was discovered by the Hastings lab working on luminescence of *A. fischeri* [10,11] and subsequently the genetic basis was unravelled leading to the characterization of the two major protein components AHL synthase (LuxI) and the AHL receptor (LuxR) [12,13].

Vibrionaceae are not only important as symbiotic or pathogenic bacteria, but have more recently also been hailed as a potential source of novel bioactive secondary metabolites [14,15,16,17] such as the antibiotic molecules andrimid and holomycin [18], the antifungal compound vibrindole A [19] and the anticancer-active pelagiomicin C [20]. Furthermore, *Photobacterium* *halotolerans* produces solonamides and ngercheumicins that interfere with quorum sensing and virulence in *Staphylococcus aureus* [21,22]. The QS-interfering *Photobacterium* sp. and the antibiotic producing *Vibrio* spp. were isolated during a screening of 500 bacterial strains collected on the global marine research cruise Galathea 3 [23]. Three hundred and one of the 500 strains were identified as Vibrionaceae by 16S rRNA gene analysis and are the subject of this study.

QS molecules have been detected in several symbiotic and pathogenic *Vibrionaceae* species, including *V. harveyi* [24], *V. cholerae* [25], *V. anguillarum* [26,27], *A. salmonicida* [28], *V. vulnificus* [29] and *Photobacterium phosphoreum* [30]. In some species, the phenotypes regulated by QS have been determined, e.g., in *A. fischeri*, QS controls approximately 25 genes, including those for light production [13,31]. Yang *et al.* surveyed a collection of *Vibrio* type strains, the nomenclatural representatives of a species, obtained from culture collections using biological AHL monitors [9]. They found that 20 of 24 different Vibrionaceae species produced compounds that elicited the AHL monitors. Based on the thin-layer chromatography profile and response to a biomonitor, they concluded that *N*-hexanoyl (C6), *N*-octanoyl (C8), *N*-(3-oxo-hexanoyl) (O-C6) and *N*-(3-oxo-octanoyl) (O-C8) homoserine lactones (HLs) were found, however, chemical confirmation was not included in the study [9]. García-Aljaro *et al.* developed a double-layer microplate high-throughput assay for testing 106 *Vibrio* spp. isolates [32]. Twenty of the 28 species were identified by 16S rRNA and were able to induce a response in the AHL biomonitors. Later, Purohit *et al.* detected AHLs in about half of the 57 strains from the genera *Aliivibrio*, *Photobacterium* and *Vibrio* that they screened for AHLs [33]. They searched for 15 different AHLs using HPLC-MS/MS finding *N*-hexanoyl (C6), *N*-octanoyl (C8), *N*-(3-hydroxy-decanoyl) (OH-C10) and *N*-(3-oxo-hexanoyl) (O-C6) homoserine lactones as the dominant AHLs.

In this study, we investigated the abundance and diversity of AHLs in 301 Vibrionaceae strains from a global collection [23]. The purpose of this survey was to determine how widespread AHL signalling is in environmental Vibrionaceae spanning most climate zones using biological monitors as well as LC-MS identification. The study is part of a larger work in which we aim to determine to what degree QS is involved in regulating production of bioactive secondary metabolites in Vibrionaceae.

## 2. Results and Discussion

### 2.1. Method Performance

Typically, *A. tumefaciens* detects regular, 3-OH- and O-HLs and is especially sensitive to longer chain AHLs (>C4) [34], while *C. violaceum* specifically reacts to short chain AHLs (C4 to C8) [35]. To confirm as well as extend the current knowledge on the AHL detection ability of these strains in a standardized manner, pure standard solutions of C4, C6, C8, C10, C12, C14, C18, OH-C4, OH-C6, OH-C12, O-C4, O-C6, O-C8, O-C10, and O-C12 homoserine lactones were evaluated in both biomonitor assays in three 10-fold dilutions. All AHLs resulted in a response in at least one of the assays (Supplementary Table S1). For *A. tumefaciens*, the highest response was detected for O-C8, O-C10, O-C12, C6, C8, C10, OH-C6, and OH-C12. No response was detected with C4 (4089 µmol·L^−1^) (the highest dose tested is given in parentheses). *C. violaceum* responded to all AHLs, except C14 (3211 µmol·L^−1^), C18 (2721 µmol·L^−1^) and O-C12 (336 µmol·L^−1^) homoserine lactone. However, except for C4, C6, and the highest concentrations of O-C4 (5400 µmol·L^−1^), O-C6 (47 µmol L^−1^) and O-C8 (41 µmol L^−1^) homoserine lactone, the responses of *C. violaceum* were rather weak. These results confirm that *A.* *tumefaciens* reacts to all types of AHLs, including four previously untested AHLs (O- and OH-C4, C14 and C18), but is generally more sensitive to AHLs longer than C4 up to C18. A response in *C. violaceum* is induced by all types of AHLs with a maximum chain length of C12, including the previously untested oxo-HLs (O-C4 to O-C12).

Limit of detection (LOD, after re-extraction from growth media) for the different assays demonstrates that UHPLC-HRMS (most abundant ion ± *m/z* 0.01 [M + H]^+^ or [M + Na]^+^ with signal/noise 1:10) was the most sensitive method for most compounds except for the long chained oxo AHLs (Table 1). 

**Table 1:** Limit of detection (nmol L^−1^) of a subset of acyl homoserine lactones (AHLs) using the three different assays. ND = not detected.

Assay	Limit of Detection (nmol L^−1^)							
	C4	C6	C8	C10	O-C6	O-C8	O-C10	OH-C6
UHPLC-HRMS ^a^	40	25	30	70	25	150	125	14
*C. violaceum* ^b^	10,200	250	220	15,700	235	200	1850	230
*A. tumefaciens* ^b^	ND	12,600	22	200	25	10	2	230

^a ^Concentration in spiked medium extracted by ethyl acetate as described in the text (using small molecule MS tuning); ^b ^From pure standard solutions recalculated to medium concentration assuming 100% recovery.

Compared to Purohit *et al.* [33], the LC-MS LODs reported here are 3–70 times higher which is to be expected when comparing a Time of Flight MS against the more sensitive triple quadrupole MS. The method herein presented can identify unexpected AHLs, enable retrospective data-analysis, and tentatively identify AHLs. Furthermore, it is more specific due to more fragment ions being detected, high resolution determination of the fragment ions, and high resolution determination of several pseudomolecular ions ([M + H]^+^, [M + Na]^+^ and [M + NH_4_]^+^).

**Figure 1:**
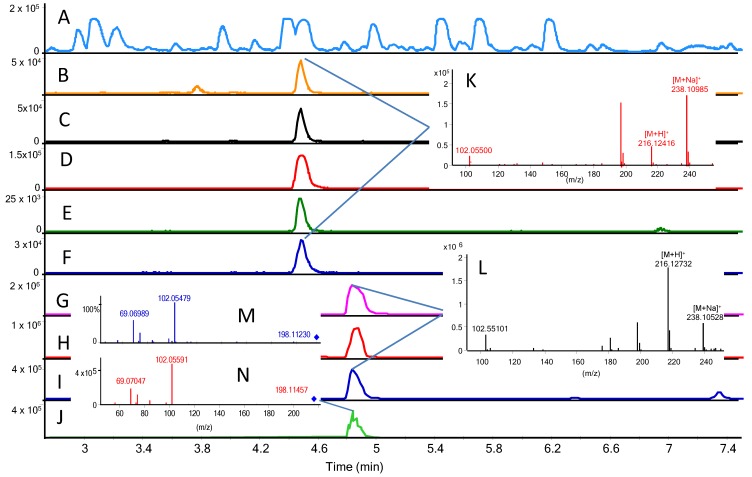
Detection of OH-C6 in S0203 using two different methods: **A**–**F** (standard tune) and **G**–**J** (small molecule tune). (**A**) BPC chromatogram; (**B**–**F**) extracted ion chromatograms of [M + H]^+^ ±0.5, [M + H]^+^ ±0.01, [M + Na]^+^ ±0.01, 102.05495 ±0.01, and 1 × ^13^C [M + Na]^+^ ±0.01; (**G**–**I**) extracted ion chromatograms of [M + H]^+^ ±0.5, [M + H]^+^ ±0.01, 102.05495 ±0.01, and [M + Na]^+^; (**J**) MS/HRMS trace of 216.123. M: MS/HRMS Library spectrum of OH-C6 [M + H]^+^ at 10 eV, N: simultaneously acquired MS/HRMS spectrum at 10 eV; (**L**) full scan spectrum (small molecule tune); (**K**) full scan spectrum (normal tune). Samples were analysed with a time difference of two months and thus retention was altered, but constant in the sequence and identical (±0.02 min) to reference standard.

At concentrations 2–3 above LOD (if only using one ion), qualifier ions could additionally be detected by giving increasing strength to the identification (Figure 1B–J). In Figure 1B–F,K, the default tuning was used, which results in [M + H]^+^ being less predominant than [M + Na]^+^ as [M + H]^+^ are lost due to fragmentation, while [M + Na]^+^ is more stable (and cannot be used for MS/MS experiments). In Figure 1G–J,L, the data from small molecule tuning is shown, providing much more [M + H]^+^, which can be MS/MS fragmented into a very specific fingerprint (Figure 1N) that is identical with the library spectrum seen (Figure 1M). Thus, it provides an additional identification method for the AHLs via their tandem HRMS spectra [36]. These MS/MS spectra are less noisy than full scan spectra, as all ions except the parent ion (±*m/z* 0.5) are filtered away; therefore, all fragment ions are generated from the parent ion. In this case, by using a QTOF MS, the fragment ions are additionally measured with high mass accuracy (MS/HRMS). Due to the losses during the MS/MS fragmentation and that the [M + H]^+^ and/or [M + NH_4_]^+^ needs to be auto-selected for MS/MS, (this analysis is not as sensitive as the full scan shown in Figure 1B–J). Nonetheless, the MS/HRMS sensitivity was in almost all cases sensitive enough for the detection of AHLs.

More importantly, the MS/HRMS spectra of the reference standard AHLs showed the known fragment ions *m/z* 102.05495 (homoserine ring) and C_3_H_5_N, *m/z* 56.04948 (further loss of CH_2_O_2_ from the homoserine ring) as well as *m/z* 120.06552 derived from the open homoserine ring moiety. Thus, data files could be mined for open and closed form AHLs by extracted ion chromatograms of *m/z* 102.05495, 56.04948, 120.06552 (±*m/z* 0.01) from all MS/HRMS precursors. This is similar to parent ion scanning on triple quadruple instruments, and can identify unexpected AHLs as long as they contain the homoserine ring moiety.

### 2.2. AHL-Producing Vibrionaceae Strains

Classification of the bacterial isolates as Vibrionaceae was confirmed by 16S rRNA gene sequencing (GenBank acc. no. in Table 2). Additionally, all strains grew well on TCBS-agar confirming their *Vibrio*-specific metabolism. Of the 301 strains, 32 (corresponding to 11%) induced a response in either *A. tumefaciens* or *C. violaceum* when *Vibrio* biomass from marine agar was spotted directly onto reporter plates (Table 3). Nine strains induced both monitors, while 15 induced only the *A. tumefaciens* and eight only the *C. violaceum*.

**Table 2:** 32 AHL-producing Vibrionaceae strains and their taxonomic identification by multilocus sequences analysis (MLSA) of the 16S rRNA, *recA*, *toxR* and *rpoA* gene sequences and geographic data of isolation site.

Strain	16S rRNA GenBank Acc. No.	Closest Relative by MLSA	*fur* Gene Analysis		Isolation Site	
			Species	E Value	Latitude	Longitude
S0188	FJ457302	*V. lentus* ^a^	*V. splendidus*	0	62.03815	−9.99592
S0202	FJ457304	*V. anguillarum* ^a^	*V. anguillarum* ^b^	0	62.03815	−9.99592
S0203	FJ457305	*V. lentus* ^a^	*V. splendidus*	0	62.03815	−9.99592
S0207	KM273118	*V. anguillarum*	*V. anguillarum* ^b^	0	62.16527	−16.5731
S0209	FJ457309	*Vibrio* sp.	*V. splendidus*	0	62.16527	−16.5731
S0273	FJ457310	*V. lentus* ^a^	*V. splendidus*	0	62.2569	−20.8819
S0344	FJ457313	*V. pacinii* ^a^	*V. pacinii*	0	66.7494	−53.8954
S0787	FJ457334	*V. mediterranei* ^a^	*V. shilonii*	0	33.7571	−25.4239
S0821	FJ457339	*V. furnissii*	*V. fluvialis*	0	23.0918	−24.0417
S0843	FJ457343	*V. brasiliensis*	*V. brasiliensis*	0	12.20592	−21.0234
S0845	FJ457345	*V. brasiliensis*	*V. brasiliensis*	0	12.20592	−21.0234
S1073	FJ457350	*V. nigripulchritudo*	*V. nigripulchritudo*	0	1.6207	−10.5021
S1089	FJ457355	*V. campbellii*	*V. campbelli*	0	4.570467	−1.72975
S1106	FJ457363	*V. anguillarum*	*V. anguillarum* ^b^	0	4.570467	−1.72975
S1110	FJ457366	*V. fluvialis*	*V. fluvialis*	0	4.570467	−1.72975
S1137	FJ457371	*V. chagasii* ^a^	*V. splendidus*	9.32E−158	4.9119	−0.3376
S1162	FJ457375	*V. fluvialis*	*V. fluvialis*	0	4.570467	−1.72975
S1192	KM273119	*Photobacterium angustum* ^a^	ND		4.570467	−1.72975
S1194	KM273120	*V. campbellii*	*V. campbelli*	0	4.570467	−1.72975
S1196	KM273121	*V. campbellii*	*V. campbelli*	0	4.570467	−1.72975
S1728	FJ457408	*V. anguillarum*	*V. anguillarum* ^b^	2.52E−170	−19.7461	114.8573
S1729	FJ457409	*V. anguillarum*	*V. anguillarum* ^b^	0	−19.7461	114.8573
S1730	FJ457410	*V. anguillarum*	*V. anguillarum* ^b^	0	−19.7461	114.8573
S1732	FJ457411	*V. anguillarum*	*V. anguillarum* ^b^	0	−19.7461	114.8573
S2605	FJ457458	*V. brasiliensis* ^a^	*V. tubiashii*	3.02E−144	−10.3454	157.7956
S2606	FJ457459	*V. brasiliensis* ^a^	*V. tubiashii*	3.02E−144	−10.3454	157.7956
S2719	FJ457471	*Vibrio* sp.	*V. brasiliensis*	2.35E−154	−8.1005	156.8451
S2757	FJ457478	*Vibrio* sp.	*V. tubiashii*	2.88E−170	−15.2329	156.665
S3857	FJ457565	*Vibrio* sp.	*V. rotiferianus*	0	−14.159	−77.4004
S4497	FJ457596	*Vibrio* sp.	*V. rotiferianus*	0	24.9963	−67.0246
S4634	FJ457597	*V. splendidus*	*V. splendidus*	3.25E−116	43.0309	−66.2774
S4738	FJ457608	*Vibrio* sp.	*V. splendidus* ^b^	0	58.8041	−3.0564

^a ^MLSA was uncertain; ^b ^sequenced contig BLASTed against NCBI, not the *fur* gene database; ND = not defined.

**Table 3:** AHLs in 32 Vibrionaceae strains tested against *C. violaceum* (*Cv*) and *A. tumefaciens* (*At*) using biomass or acidified EtOAc extracts and AHLs detected by UHPLC-DAD-QTOFMS; numbers demonstrate the peak area of the AHL in the chromatogram of the first run; numbers in brackets demonstrate the peak area of the AHL in the chromatogram of the second run; the total no. of occurrences/AHL does not include the reference strain *V. anguillarum* 90-11-287.

Strain	AHL Response in				No of AHL/Strain	Peak Area (1000 Counts) of AHL in Chromatogram																				
	Biomass Screen		Extract Well Assay																							
	*Cv*	*At*	*Cv*	*At*		C4	C6	C7	C8	C12	OH-C4	OH-C6	OH-C4	OH-C6	OH-C9	OH-C10	OH-C11	OH-C12	OH-C14	O-C4	O-C6	O-C8	O-C9	O-C10	O-C11	O-C12
S0188 ^a^	−	+	+	+	9	ND	(114) ᶜ	178 ᵇ	ND	ND	32 ^b^ (161) ^b^	880 ^b^ (4822) ^b^	32 ^b^ (161) ^b^	880 ^b^ (4822) ^b^	ND	46^ c^ (561) ^b^	ND	12^ c^ (163)^ b^	(21313)^ b^	ND	ND	ND	ND	ND	ND	ND
S0202	−	+	−	+	5	ND	ND	ND	ND	ND	ND	118 ^c^ (1659) ^b^	ND	118 ^c^ (1659) ^b^	ND	(112)^ b^	ND	ND	ND	ND	ND	(182)^ b^	ND	90^ c^ (3385)^ b^	ND	(30)^ b^
S0203 ^a^	−	+	+	+	7	ND	ND	ND	ND	ND	40 ^b^ (3709) ^b^	964 ^b^ (6479) ^b^	40 ^b^ (3709) ^b^	964 ^b^ (6479) ^b^	ND	58^ c^ (412)^ b^	ND	16^ c^ (256)^ b^	(94)^ b^	ND	ND	ND	ND	ND	ND	ND
S0207	+	+	−	+	4	ND	ND	ND	ND	ND	ND	167 ^c^ (1807) ^b^	ND	167 ^c^ (1807) ^b^	ND	(70)^ b^	ND	ND	ND	ND	ND	ND	ND	96^ c^ (3637)^ b^	ND	(43)^ b^
S0209 ^a^	+	+	+	+	8	ND	(116) ᵇ	ND	ND	ND	17 ^c^ (342) ^b^	627 ^b^ (4297) ^b^	17ᶜ (342) ^b^	627 ^b^ (4297) ᵇ	(30)^ b^	28^ c^ (452)^ b^	ND	(64)^ b^	ND	ND	ND	ND	ND	ND	ND	ND
S0273 ^a^	+	+	+	+	7	ND	ND	ND	ND	ND	28 ^b^ (273) ^b^	896 ^b^ (5918) ^b^	28 ^b^ (273) ^b^	896 ^b^ (5918) ^b^	ND	75^ c^ (485)^ b^	ND	17^ c^ (278)^ b^	(115)^ b^	ND	ND	ND	ND	ND	ND	ND
S0344	+	−	−	−	2	92 ^b^ (5352) ^b^	ND	ND	ND	ND	(50)ᵇ	ND	(50)ᵇ	ND	ND	ND	ND	ND	ND	ND	ND	ND	ND	ND	ND	ND
S0787	−	+	−	+	0	ND	ND	ND	ND	ND	ND	ND	ND	ND	ND	ND	ND	ND	ND	ND	ND	ND	ND	ND	ND	ND
S0821	+	+	+	+	6	ND	ND	ND	57 ^b^ (6284) ^b^	ND	ND	ND	ND	ND	ND	ND	ND	ND	ND	ND	ND	(294)^ b^	11^ c^ (423)^ b^	(2991)^ b^ 82^ c^	10^ c^	(72)^ b^
S0843 ^a^	+	−	+	+	2	317 ^b^ (11974) ^b^	ND	ND	ND	ND	(94)ᵇ	ND	(94)ᵇ	ND	ND	ND	ND	ND	ND	ND	ND	ND	ND	ND	ND	ND
S0845	+	−	−	−	1	83 ^b^ (5009) ^b^	ND	ND	ND	ND	ND	ND	ND	ND	ND	ND	ND	ND	ND	ND	ND	ND	ND	ND	ND	ND
S1073 ^a^	−	+	−	+	0	ND	ND	ND	ND	ND	ND	ND	ND	ND	ND	ND	ND	ND	ND	ND	ND	ND	ND	ND	ND	ND
S1089	−	+	−	+	4	ND	ND	ND	ND	(125322) ^b^	310 ^b^ (2279) ^b^	(119) ^b^	310 ^b^ (2279) ^b^	(119) ^b^	ND	ND	ND	42^ c^ (1313)^ b^	ND	ND	ND	ND	ND	ND	ND	ND
S1106	+	+	−	+	4	ND	ND	ND	ND	ND	ND	140 ^c^ (2050) ^b^	ND	140 ^c^ (2050) ^b^	ND	(93)^ c^	ND	ND	ND	ND	ND	ND	ND	86^ c^ (3661)^ b^	ND	(41)^ b^
S1110	+	+	−	+	2	ND	ND	ND	ND	ND	ND	ND	ND	ND	ND	ND	ND	ND	ND	ND	ND	ND	ND	58^ c^ (2770)^ b^	(305)^ b^	ND
S1137	+	+	−	+	2	ND	ND	ND	ND	ND	ND	210 ^c^ (3154) ^b^	ND	210 ^c^ (3154) ^b^	ND	ND	ND	ND	ND	ND	ND	ND	ND	ND	ND	ND
S1162	−	+	−	+	8	ND	ND	ND	ND	ND	ND	ND	ND	ND	ND	239^ c^ (134)^ b^	59^ b^ (503)^ b^	ND	ND	30^ b^	ND	12^ c^ (273)^ b^	31^ c^ (620)^ b^	241^ c^ (5862)^ b^	623^ b^ (10784)^ b^	12^ c^
S1192	−	+	−	+	1	ND	ND	ND	ND	ND	ND	ND	ND	ND	ND	ND	ND	(104)^ b^	ND	ND	ND	ND	ND	ND	ND	ND
S1194	−	+	−	−	2	ND	ND	ND	ND	ND	61 ^b^ (491) ^b^	ND	61 ^b^ (491) ^b^	ND	ND	ND	ND	(215)^ b^	ND	ND	ND	ND	ND	ND	ND	ND
S1196	−	+	−	+	2	ND	ND	ND	ND	ND	163 ^b^ (1425) ^b^	ND	163 ^b^ (1425) ^b^	ND	ND	ND	ND	ND (80)^ b^	ND	ND	ND	ND	ND	ND	ND	ND
S1728	−	+	−	+	4	ND	ND	ND	ND	ND	ND	61 ^c^ (617) ^b^	ND	61 ^c^ (617) ^b^	ND	(78)^ b^	ND	ND	ND	ND	ND	ND	ND	89^ c^ (3069)^ b^	ND	(106)^ b^
S1729	−	+	+	+	7	ND	ND	ND	53 ^b^ (5170) ^b^	ND	ND	268 ^c^ (2943) ^b^	ND	268 ^c^ (2943) ^b^	ND	17^ b^ (124)^ b^	ND	ND	ND	ND	(303)^ b^	11^ c^ (293)^ b^	ND	171^ c^ (6066)^ b^	ND	16^ c^ (77)^ b^
S1730	−	+	−	+	5	ND	ND	ND	ND	ND	ND	142 ^c^ (2643) ^b^	ND	142 ^c^ (2643) ^b^	ND	(147)^ b^	ND	ND	ND	ND	ND	(274)^ b^	ND	115^ c^ (5836)^ b^	ND	(52)^ b^
S1732	−	+	−	+	5	ND	ND	ND	ND	ND	ND	201ᶜ (2201) ᵇ	ND	201 ^c^ (2201) ^b^	ND	12^ c^ (100)^ b^	ND	ND	ND	ND	ND	10^ c^ (233)^ b^	ND	149^ c^ (5687)^ b^	ND	(68)^ b^
S2605	+	−	−	+	1	ND	ND	ND	ND	ND	ND	33^c^ (369) ^b^	ND	33 ^c^ (369) ^b^	ND	ND	ND	ND	ND	ND	ND	ND	ND	ND	ND	ND
S2606 ^a^	+	−	+	+	6	ND	(773) ^c^	806 ^b^	ND	ND	(90)^b^	961^b^ (6248) ^b^	(90)^b^	961 ^b^ (6248) ^b^	ND	ND	ND	ND	ND	ND	ND	ND	ND	ND	ND	ND
S2719	+	−	−	+	2	77 ^b^ (4578) ^c^	(54) ^c^	ND	ND	ND	ND	ND	ND	ND	ND	ND	ND	ND	ND	ND	ND	ND	ND	ND	ND	ND
S2757	+	+	+	+	3	ND	(69) ^c^	ND	ND	ND	ND	364 ^c^ (5464) ^b^	ND	364^c^ (5464) ^b^	ND	ND	ND	ND	ND	ND	(220)^ b^	ND	ND	ND	ND	ND
S3857	+	+	+	+	2	ND	52 ^c^ (5321) ^b^	ND	(768) ^b^	ND	ND	ND	ND	ND	ND	ND	ND	ND	ND	ND	ND	ND	ND	ND	ND	ND
S4497 ^a^	+	−	+	+	3	19 ^b^ (917) ^b^	286 ^b^ (12817) ^b^	631 ^b^	ND	ND	ND	ND	ND	ND	ND	ND	ND	ND	ND	ND	ND	ND	ND	ND	ND	ND
S4634	+	−	−	+	1	ND	ND	ND	ND	ND	ND	(148)^b^	ND	(148) ^b^	ND	ND	ND	ND	ND	ND	ND	ND	ND	ND	ND	ND
S4738 ^a^	−	+	−	+	1	ND	ND	ND	ND	ND	219 ^b^ (2638) ^b^	ND	219 ^b^ (2638) ^b^	ND	ND	ND	ND	ND	ND	ND	ND	ND	ND	ND	ND	ND
90-11-287	−	+	+	+	3	ND	ND	ND	ND	ND	ND	192 ^c^ (2162) ^b^	ND	192 ^c^ (2162) ^b^	ND	(46)^ c^	ND	ND	ND	ND	ND	ND	ND	111^ c^ (3228)^ b^	ND	ND
**No of occurrence/AHL**						5	7	3	3	1	11	17	11	17	1	12	1	8	3	1	2	6	2	11	3	9

^a ^strains grown in LB20; ^b ^AHL was identified by multiple adduct ions; ^c ^AHL was identified only as [M + H]^+^ or [M + Na]^+^; ND = not detected.

Using five different reporter strains in a double-layer microplate high-throughput assay, 85% of Vibrionaceae isolates gave a positive response in a study by García-Aljaro *et al.* [32]. Without using a biomonitor prescreen, Purohit *et al.* [33] analysed 57 Vibrionaceae isolates for AHLs by HPLC-MS/MS and did not recover AHLs from nine of the strains, also demonstrating a hit rate of about 85% [33]. Our comparably low number of AHL-positive strains could be correlated with the lower number of biomonitor strains, our pre-screening method or the strain diversity. However, an increased number of biomonitor strains has not yielded an improved detection of AHLs in Vibrionaceae previously [32]. The media used in the bioassays have low salt concentrations, which could have caused stress to the spotted Vibrionaceae biomass and thus could lead to an underestimate of the total number of AHL-positive strains. Hence, the herein AHL-negative strains may still be AHL-producers in the environment or under different culturing conditions including variation in nutrients, temperature, salinity or pH. Also, they may require specific biological cues from other organisms to trigger the production of QS signals. These variations and optimizations might be included in future studies to gain more knowledge on the physiological background of AHL production in Vibrionaceae. The strain collections used in the two studies described above [32,33] were selected strains, namely fish-derived strains. Our strain collection represents a broader range of environmental strains and this difference in strain profile could also be the cause of the differences in AHL-positive strains.

For extract preparation, the 32 AHL-positive strains were grown in LB10. Nine strains (S0188, S0203, S0209, S0273, S0843, S1073, S2606, S4497 and S4738) did not grow in LB10 and required higher salinity and thus, they were grown in LB20 (Table 3). All ethyl acetate extracts were re-tested for induction of the monitors *A. tumefaciens* and *C. violaceum* in a well assay. Extracts of 15 strains demonstrated a different response compared to the initial biomass screen, either by gaining or loosing activity for one of the biomonitors. This could be attributed to the change of bacterial growth condition (plate *versus* liquid medium) or the final concentration of AHLs in the extracts, which may or may not have crossed the limit of detection [34,35]. In *V. anguillarum* 90-11-287, a positive reaction for C6-AHL was observed in the biomonitor strain as evaluated with biomass, while LC-MS did not detect C6-AHL [26]. False positives might potentially occur when a strain is spotted too close to another strain on a plate. In this second screen, 10 extracts induced both monitors, while 18 induced only the *A. tumefaciens* and interestingly, none of the extracts induced *C. violaceum* alone. The higher hit rate for *A. tumefaciens* has been described before and is probably due to the wider range of response to various AHLs of this strain [32] (see Section 2.1).

Three extracts (of strains S0344, S0845, S1194) were negative in the bioassays. However, in all three extracts, AHLs were detected by LC-MS, namely either *N*-butanoyl (C4), *N*-(3-hydroxy-butanoyl) (OH-C4) or *N*-(3-hydroxy-hexanoyl) (OH-C6) HL (Table 3). *A. tumefaciens* is not sensitive to these short chain AHLs, however *C. violaceum* does react at least to C4 homoserine lactone (see Section 2.1). Comparing the chemical analyses of all extracts in standard and small molecule tune, the sizes of the peak areas indicate that the concentration of AHL in these three samples might not have been sufficient for detection by the biomonitor.

### 2.3. Structural Abundance of AHLs in Vibrio Strains

UHPLC-DAD-QTOFMS was performed on extracts of the 32 strains and control strain *V. anguillarum* 90-11-287 to identify the AHL molecules produced. *V. anguillarum* 90-11-287 produced three AHLs, *N*-(3-hydroxy-hexanoyl) (OH-C6), *N*-(3-hydroxy-decanoyl) (OH-C10) and *N*-(3-oxo-decanoyl) (O-C10) homoserine lactone, agreeing with previous studies [26,33]. Across all strains, a total number of 21 different AHLs was detected (Table 3) of the 33 closed ring forms searched for. A few open ring forms were also detected, however, only in very small amounts, since open ring forms are generally closed by the extraction procedure. The maximum number of nine different AHLs was found in *V. splendidus* S0188. It is known that several Vibrionaceae strains, e.g., *V. anguillarum*, produce a number of AHLs, however, production of nine AHLs appears to be unique for a single strain.

It is important to mention that we have used a very sensitive chemical detection procedure, thus this type of diversity might be found in other organisms, but goes currently undetected. The high number of AHLs might result from biochemical processes inside the cells and might not be genetically encoded or biologically relevant in environmental conditions. However, they could potentially be part of differentiated signaling at the species level. The genome of this strain will be subjected to sequence analysis in the future. Eighteen strains produced three AHLs or more, and in five strains only one type of AHL was identified. In two strains (S0787, 1073), no AHLs could be identified from the full scan MS data, nor from the MS/MS data (e.g., *m/z* 102 fragment), although for both, monitor response was observed. This might indicate that novel unknown AHLs (with modifications on the homoserine ring that would not create the *m/z* 102 fragment) might be produced by these strains or that other types of molecules, such as diketopiperazines, are being produced [37]. This will be studied in more detail in the future including accurate concentration measurements of the detected AHLs.

**Figure 2:**
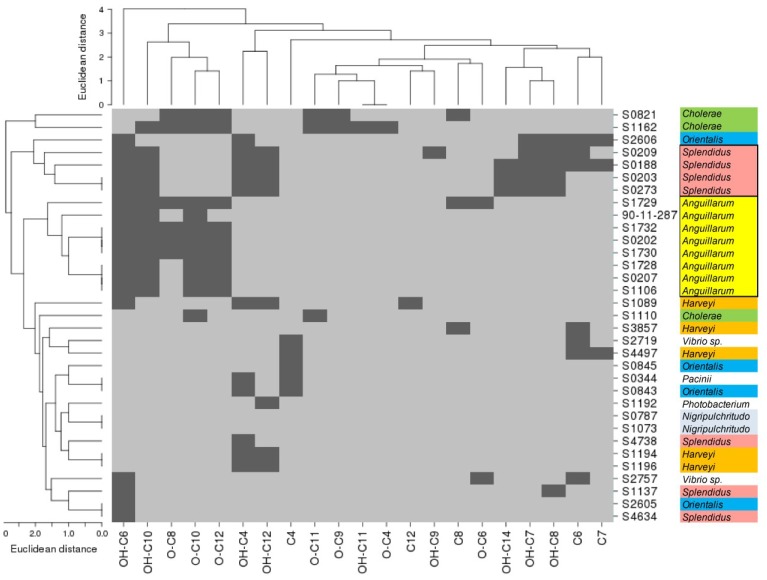
Cluster analysis of AHL diversity per strain and phylogenetic affiliation using CIMminer [43]. Dark grey: AHL present; light grey: AHL absent; colours distinguish different clades, clusters marked in bold.

The most abundant AHL was OH-C6 homoserine lactone, which was identified in 17 extracts, followed by OH-C10, OH-C4 and O-C10 homoserine lactone, which were identified in 12, 11 and 10 extracts, respectively. Rare AHLs were C12, OH-C9, OH-C11 and O-C4. The Vibrionaceae extracts studied by Purohit *et al.* [33] were dominated by O-C6, OH-C10 and OH-C4, demonstrating a certain overlap. As indicated above, variations might be due to the different species composition in our strain collection. For the strains belonging to the *Splendidus* cluster, they detected C4, OH-C4, OH-C6, OH-C8 and O-C6, while we measured OH-C4, OH-C6, OH-C7, OH-C8, OH-C10 and OH-C12, a pattern conserved through four different isolates (Figure 2). Variations could be due to the differences in growth conditions or chemical analysis applied; also for example, Purohit *et al.* [33] did not scan for odd-numbered AHLs. Generally, AHLs with odd numbers of carbons are rare in nature [38], however, they are known to be produced by other gammaproteobacteria such as *Pseudomonas aeruginosa* [39], *Yersinia ruckeri* [40] or *Edwardsiella tarda* [41]. Furthermore, *Aliivibrio fischeri* has been to shown to be capable of detecting odd-numbered AHLs such as C5 [42]. To the authors’ knowledge, this is the first study demonstrating the production of odd-numbered AHLs by strains of the Vibrionaceae family.

### 2.4. MS/HRMS Screening of AHLs

**Figure 3:**
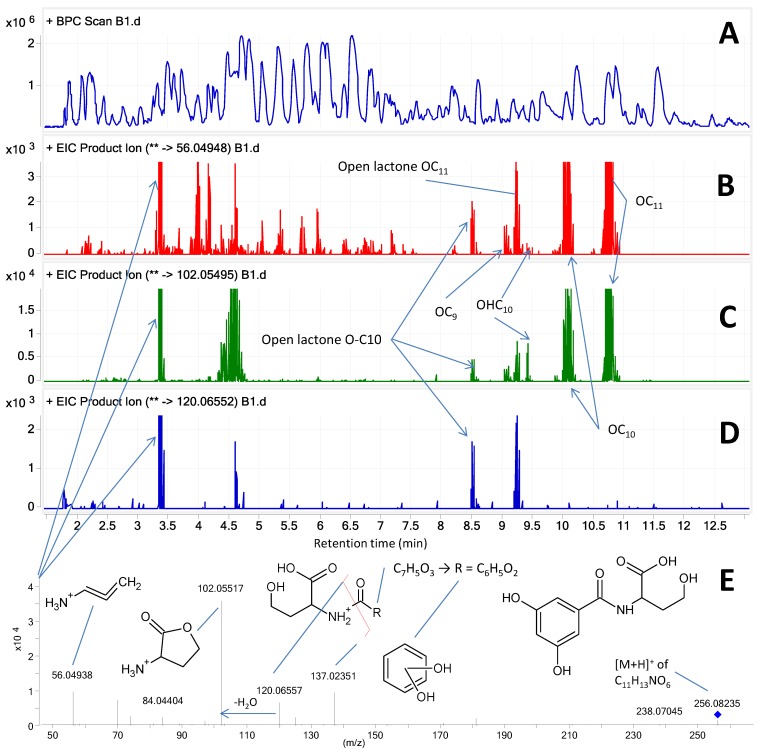
Chemical analysis of strain S1162. (**A**) base peak chromatogram; (**B**) *m/z* 56.04948 ± 0.02; (**C**) *m/z* 102.05495 ± 0.02; and (**D**) *m/z* 120.06552 ± 0.02, showing the extracted ion chromatograms of the three major diagnostic fragment ions with labels of six known long chain AHLs. At 3.36 min a novel open chain AHL is eluting with its MS/HRSM spectrum at 10 eV showed in E with a tentatively identified compound.

The upper base peak chromatogram (BPC, *m/z* 50–1700) (Figure 3A) of the ethyl acetate extract from strain S1162 demonstrates the complexity of the sample, with B–D showing the extracted ion chromatograms of the major AHL fragment ions (Figure 3); clearly indicating the presence of two unassigned peaks at 4.7 and 3.36 min. The first peak was also present in sample blanks, while the second peak corresponded to an open lactone AHL. The peaks at *m/z* 120 and 137 correspond to fragmentation between the homoserine and alkyl chains with the charge residing on one or the other side. With the fragment at *m/z* 137 containing a C=O group (where the charge is believed to reside), the accurate mass left exactly C_6_H_5_O_2_ requiring four unsaturations. This is most likely possible using an aromatic ring containing two presumed phenolic groups, although a larger ring with ketones cannot be excluded either. Nonetheless, this compound seems to be novel AHL, a finding that needs to be confirmed by preparative purification and NMR techniques or total synthesis. Bioinformatic analysis of the PKS genes in the AHL gene cluster may also aid in this tentative identification of the molecule and genome sequencing is in progress.

### 2.5. Phylogeny and Geographical Distribution of AHL-Producing Strains

**Figure 4:**
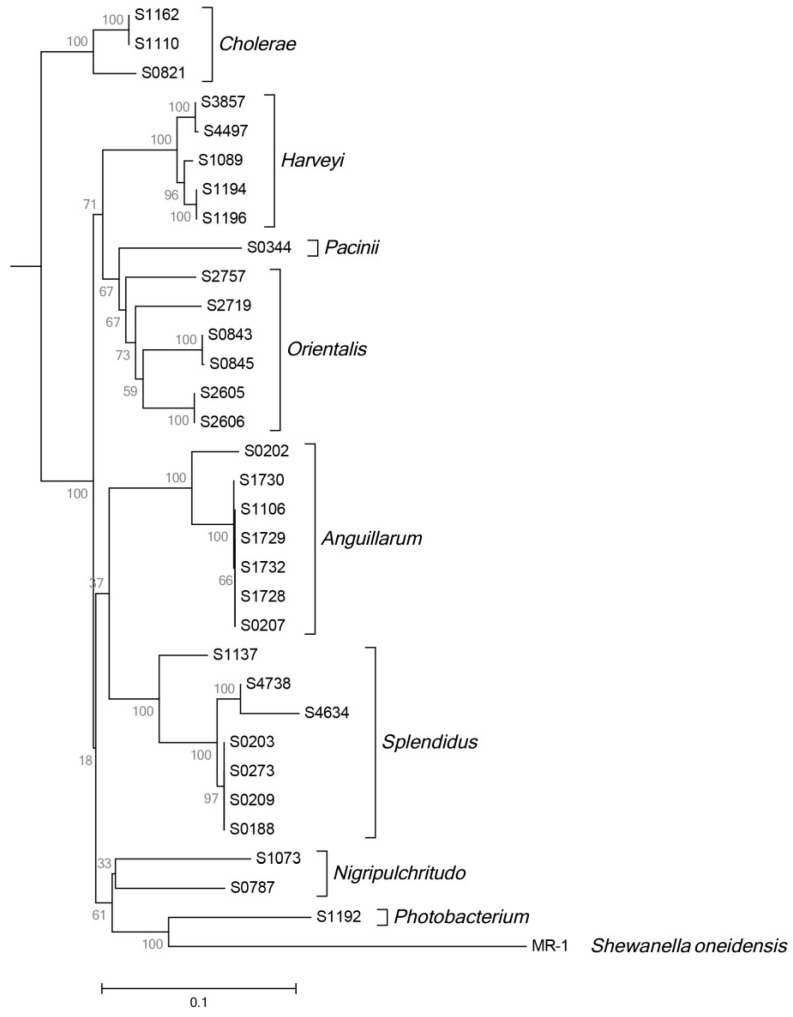
Neighbor joining tree of concatenated partial *rpoA* and *recA* gene sequences of 32 AHL-positive Vibrionaceae strains using the Jukes-Cantor method and *Shewanella oneidensis* MR-1 as outgroup. Bootstrap values are based on 100 replicates. Square brackets indicate clades [45,48].

The 32 AHL-producing strains were identified using MLSA of the 16S rRNA, *recA*, *toxR* and *rpoA* genes in combination with a novel technique using the *fur* gene for species differentiation in the family of Vibrionaceae [44]. 16S rRNA gene sequences do not allow sufficient discrimination in the Vibrionaceae family due to the presence of multiple alleles and MLSA requires the amplification of several genes. Thus, Machado *et al.* [44] demonstrated that the *fur* gene, encoding a ferric uptake regulator, would have the power to replace the previously used techniques as efficient and accurate phylogenetic marker in this family. The neighbor joining tree of concatenated partial *rpoA* and *recA* gene sequences reveals the grouping of the strains into eight Vibrionaceae clades as defined by Sawabe *et al.* [45]—namely *Cholerae*, *Harveyi*, *Pacinii*, *Orientalis*, *Anguillarum*, *Splendidus* and *Nigripulchritudo*—thereby covering half of the *Vibrio* clades (Figure 4). Additionally, we analysed one *Photobacterium* strain, *P. angustum* S1192. Within a clade, species tend to form certain phylogenetic pairs that are very difficult to distinguish, such as *V. fluvialis* and *V. furnissii*, *V. brasiliensis* and *V. tubiashii* and *V. splendidus* and *V. lentus* [46,47]. Those pairs could not be properly resolved by our MLSA analysis; however, the *fur* gene analysis was a very robust discriminator in these situations. For instance, S2719, S2757, S3857 and S4497 could not be identified using MLSA of the four marker genes, however, the *fur* analysis revealed their association with the *Orientalis* and *Harveyi* clades (Figure 4).

The 32 strains did not cluster taxonomically according to their isolation sites (Supplementary Figure S1); e.g., the seven *Anguillarum* strains were isolated from opposite sides of the globe: the west coast of Africa and the west coast of Australia. In addition, there is no tendency for strains to group together according to their climate zone; moreover, all *Harveyi* and all *Splendidus* clade strains were isolated from tropical waters. Finally, no correlation between isolation site and AHL profile, climate zone and abundance or diversity of AHLs was observed. 

When comparing phylogeny to the AHL profiles of the strains (Figure 2), two distinct clusters formed: as with the control strain *V. anguillarum* 90-11-287, most other *Anguillarum* clade isolates produced OH-C6, OH-C10 and O-C10 homoserine lactone, building a fingerprint for the *Anguillarum* clade. Four out of the six strains of the *Splendidus* clade produce OH-C4, OH-C6, OH-C7, OH-C8, OH-C10 and OH-C12. These results, where not every strain of the clade fell in one fingerprint, resemble the taxonomic pattern. This could mean that a fingerprint possibly represents a distinct subgroup of a clade rather than a complete clade (“subclade”). The *Anguillarum* and *Splendidus* strains were taxonomically very similar (Figure 4).

However, the *Anguillarum* clade strains were isolated from four different isolation sites from both temperate and tropical climates. The *Splendidus* clade strains were isolated from the same climate zone, but different locations. This demonstrates the differences between the isolates making them to unique strains besides taxonomic similarity. Combining the individual AHLs produced by the individual strains of a clade as identified by previous studies build similar profile to the herein described complete AHL profiles for the *Anguillarum* and *Splendidus* clade [26,33].

Previous studies on AHLs in Vibrionaceae have either not addressed the chemistry of the signals or lacked a high throughput setup [9,32,33]. All demonstrated a limited phylogenetic analysis using only the 16S rRNA gene. Because they possess several alleles of the 16S rRNA gene, Vibrionaceae require a more stringent phylogenetic analysis [46,47]. This, however, might be greatly facilitated by utilization of the *fur* gene as marker in the future [44]. Our findings will add a crucial amount of information to the current knowledge on AHLs in the family of Vibrionaceae and hereby, we propose an AHL fingerprint correlated to specific phylogenetic subclades.

## 3. Experimental Section

### 3.1. Bacterial Strains and Growth Conditions 

Three hundred and one Vibrionaceae strains were isolated on the global Galathea 3 expedition [23] on the basis of their ability to inhibit *Vibrio anguillarum* 90-11-287 [49]. Vibrionaceae strains were grown on Marine Agar plates (MA; Difco 212185) for 1–3 days at 25 °C for the initial AHL screening based on biomonitors. For chemical analyses of AHLs, the Vibrionaceae strains (Table 2) were grown in Luria-Bertani broth (LB; Difco 244620) under aerated conditions (200 rpm) at 20 °C for one day assuming stationary phase, which has been demonstrated for a selected number of strains. Strains that did not grow in LB were grown in LB with extra NaCl (2% final concentration). *Agrobacterium tumefaciens* NT1 (pZLR4) [34] and *Chromobacterium violaceum* CV026 [35] were used as reporter strains in bioassays to detect AHLs. *A. tumefaciens* was grown on ABt agar or in LB broth with 0.5% NaCl (LB5) with 20 µg mL^−1^ gentamycin and 50 mg mL^−1^ X-Gal. *C. violaceum* was grown on LB5 agar or in LB with 20 µg mL^−1^ kanamycin. *V. anguillarum* 90-11-287 was used as reference strain in the UHPLC-DAD-QTOFMS analysis [26]. All strains were stored at −80 °C in a freeze medium (30.0 g tryptone soy broth (TSB; Oxoid CM129B), 5.0 g glucose, 20.0 g skim milk powder, 40.0 g glycerol, 1000 mL H_2_O) [50].

### 3.2. Phylogenetic Analysis of Strains

The affiliation to Vibrionaceae was confirmed by 16S rRNA gene sequencing and streaking strains on *Vibrio*-selective medium, thiosulfate-citrate-bile salts-sucrose agar (TCBS; Oxoid CM0333) for 1–2 days at 25 °C. Genomic DNA was purified using the NucleoSpin Tissue kit (Macherey-Nagel, Düren, Germany). The genes *rpoB*, *recA*, *toxR* [18,46,47] and *fur* [44] were amplified by PCR and sequenced (GATC Biotech, Cologne, Germany). The nucleotide sequences were edited, then alignments and neighbor joining phylogenetic trees were generated using CLC Main Workbench 7 (CLC bio, Aarhus, Denmark), and visually improved using MEGA5.2 [51]. Nucleotide sequences were subjected to BLASTn analysis against the NCBI nucleotide collection for multilocus sequence analysis [52].

### 3.3. Detection of AHL Compounds

Reporter plates were prepared as described previously [53,54]. The Vibrionaceae strains were grown on MA and a loop of bacterial biomass was placed on bioassay plates embedded with reporter strain. 3 µL 1 µM OHHL (OH-C6) was spotted on the *A. tumefaciens* plates as a control and 3 µL 1 mM BHL (C4) was spotted on the *C. violaceum* plates. After 1 day at 25 °C, *C. violaceum* plates were inspected for purple zones due to AHL-induced violacein production. After 2 days at 25 °C, *A. tumefaciens* plates were inspected for formation of blue colour due to AHL-induced β-galactosidase activity. Extracts were prepared for all strains being positive in the pre-screen. To evaluate the extracts for AHL activity, 6 mm wells were punched in the solid bioassay plates and 30 µL extract were pipetted into the wells. Plates were read as described above.

To confirm the AHL detection range of each biomonitor, 16 AHL standards (Supplementary Table S1) were tested against *C. violaceum* and *A. tumefaciens* in a well assay, using three tenfold diluted concentrations in acetonitrile. Plates were read as described above.

### 3.4. Extracts for Bioassays and UHPLC-DAD-QTOFMS

Ten mL of liquid LB10 or LB20 culture was mixed with 10 mL ethyl acetate (EtOAc) containing 0.5% formic acid (FA), and incubated at 20 °C and 200 rpm for 30 min. The upper EtOAc phase was collected, and evaporated to dryness under nitrogen. Samples were resuspended in 0.5 mL EtOAc with 0.5% formic acid and stored at −20 °C until use. 30 µL of extracts (*i.e.*, corresponding to 1.6 mL of original culture) were re-tested for AHL activity as described above.

### 3.5. AHL Detection via UHPLC-DAD-QTOFMS

200 µL of the ethylacetate extracts were evaporated to dryness under an N_2_ atmosphere and resuspended in 200 µL 50:50 (vol/vol) Acetonitrile (ACN)-MilliQ water buffered with 20 mM FA 50:50 (vol/vol). Ultra-high performance liquid chromatography-diode array detection-quadrupole time of flight mass spectrometry (UHPLC-DAD-QTOFMS) was performed on an Agilent Infinity 1290 UHPLC system (Agilent Technologies, Santa Clara, CA, USA) with a diode array detector. An Agilent Poroshell 120 phenyl-hexyl column (2.1 × 150 mm, 2.7 µm) was used for separation with a linear gradient consisting of water and ACN both buffered with 20 mM FA and 10 mM ammonium formate, starting at 5% ACN and increased to 85% in 14, and then to 100% in 1 min where it was held for 2 min, then returned to 10% in 0.1 min and keeping it for 3 min (0.35 mL/min, 60 °C). Subsamples of 0.6 µL were injected. High resolution MS and MS/MS detection was done on an Agilent 6550 iFunnel QTOF MS [36]. MS/MS was done at 10 and 20 eV with a max of 3 parent ions and in the range *m/z* 170–460 only selecting singly charged ions, and using and ion-exclusion time of 0.04 min. Samples were analysed twice one time using the default tuning parameters and the small molecule tuning (Agilent Tune manual B6.00).

Reference standards co-analysed in each sequence were: *N*-butanoyl homoserine lactone (BHL, C4), *N*-hexanoyl homoserine lactone (HHL, C6), *N*-octanoyl homoserine lactone (OHL, C8), *N*-decanoyl homoserine lactone (DHL, C10), *N*-dodecanoyl homoserine lactone (dDHL, C12), *N*-tetradecanoyl homoserine lactone (tDHL, C14), *N*-(3-oxo-hexanoyl) homoserine lactone (OHHL, O-C6), *N*-(3-oxo-octanoyl) homoserine lactone (OOHL, O-C8), *N*-(3-oxo-decanoyl)-homoserine lactone (ODHL, O-C10), *N*-(3-oxo-dodecanoyl)-homoserine lactone (OdDHL, O-C12), *N*-(3-hydroxy-hexanoyl) homoserine lactone (OH-C6), *N*-(3-Hydroxy-decanoyl)-homoserine lactone (OH-C10), and *N*-(3-hydroxy-dodecanoyl)-homoserine lactone (OH-C10). Extracts with uneven chain length, oxo homoserine lactones, were available from a previous study [40].

Data processing was performed using MassHunter (Agilent Technologies), where MS/MS data was processed in Qualitative Analysis version B.06.00 (Agilent Technologies) while HRMS data was processed in Quantative Analysis version B.06.00 searching, using mass extraction window of *m/z* ± 0.01 of [M + H]^+^, [M + Na]^+^, [M + NH_4_]^+^, *m/z* 102.05495 (homoserine ring), *m/z* 56.04948 (homoserine ring with of CO and H_2_O or HCOOH loss), and *m/z* 120.06552 (open homoserine ring). Peaks with an s/n below 1:10 were not integrated. For evaluation of MS/HRMS data extracted ion chromatograms of the 3 fragment ions were plotted and parent ions determined manually, with a focus on scans where at least 2 of the 3 ions were detected.

## 4. Conclusions

In summary, we demonstrated that in a number of clades in the family of Vibrionaceae, a greater range of the QS signals, acyl homoserine lactones, including AHLs with odd numbers of carbon, is produced than has been previously shown. In addition to this, we have shown that these AHLs are very diverse. Our results indicate that there appears to be an AHL fingerprint for at least some of the Vibrionaceae clades. Besides the correlation of AHL profile to phylogeny, no pattern for geographic distribution was identified. This demonstrates that the genetic basis of AHLs was globally distributed during evolution, as no niche-specific AHLs were observed. This new knowledge on AHLs in Vibrionaceae will be utilized in future studies on inter- and intra-species communication.

## Supplementary Files

Supplementary File 1
