# Correction: Rasmussen, B.B., *et al.* Global and Phylogenetic Distribution of Quorum Sensing Signals, Acyl Homoserine Lactones, in the Family of Vibrionaceae. *Mar. Drugs* 2014, *12*, 5527–5546

**DOI:** 10.3390/md13031548

**Published:** 2015-03-20

**Authors:** Bastian Barker Rasmussen, Kristian Fog Nielsen, Henrique Machado, Jette Melchiorsen, Lone Gram, Eva C. Sonnenschein

**Affiliations:** 1 Department of Systems Biology, Technical University of Denmark, Matematiktorvet bldg 301, DK-2800 Kgs, Lyngby, Denmark; E-Mails: bbara@bio.dtu.dk (B.B.R.); jeme@bio.dtu.dk (J.M.); gram@bio.dtu.dk (L.G.); 2 Department of Systems Biology, Technical University of Denmark, Søltofts Plads bldg 221, DK-2800 Kgs, Lyngby, Denmark; E-Mail: kfn@bio.dtu.dk; 3 Novo Nordisk Foundation Center for Biosustainability, Technical University of Denmark, Kogle Allè 6, DK-2970 Hørsholm, Denmark; E-Mail: henma@biosustain.dtu.dk

The authors wish to make the following corrections to this paper [1]: Due to duplicated and missing data in Table 3, Page 5533, replace:

**Table 3:** AHLs in 32 Vibrionaceae strains tested against *C. violaceum* (*Cv*) and *A. tumefaciens* (*At*) using biomass or acidified EtOAc extracts and AHLs detected by UHPLC-DAD-QTOFMS; numbers demonstrate the peak area of the AHL in the chromatogram of the first run; numbers in brackets demonstrate the peak area of the AHL in the chromatogram of the second run; the total no. of occurrences/AHL does not include the reference strain *V. anguillarum* 90-11-287.

Strain	AHL Response in				No of AHL/Strain	Peak Area (1000 Counts) of AHL in Chromatogram								
	Biomass Screen		Extract Well Assay											
	*Cv*	*At*	*Cv*	*At*		C4	C6	C7	C8	C12	OH-C4	OH-C6	OH-C4	OH-C6
S0188 ^a^	−	+	+	+	9	ND	(114) ᶜ	178 ᵇ	ND	ND	32 ^b^ (161) ^b^	880 ^b^ (4822) ^b^	32 ^b^ (161) ^b^	880 ^b^ (4822) ^b^
S0202	−	+	−	+	5	ND	ND	ND	ND	ND	ND	118 ^c^ (1659) ^b^	ND	118 ^c^ (1659) ^b^
S0203 ^a^	−	+	+	+	7	ND	ND	ND	ND	ND	40 ^b^ (3709) ^b^	964 ^b^ (6479) ^b^	40 ^b^ (3709) ^b^	964 ^b^ (6479) ^b^
S0207	+	+	−	+	4	ND	ND	ND	ND	ND	ND	167 ^c^ (1807) ^b^	ND	167 ^c^ (1807) ^b^
S0209 ^a^	+	+	+	+	8	ND	(116)ᵇ	ND	ND	ND	17 ^c^ (342) ^b^	627 ^b^ (4297) ^b^	17 ᶜ (342) ^b^	627 ^b^ (4297) ᵇ
S0273 ^a^	+	+	+	+	7	ND	ND	ND	ND	ND	28 ^b^ (273) ^b^	896 ^b^ (5918) ^b^	28 ^b^ (273) ^b^	896 ^b^ (5918) ^b^
S0344	+	−	−	−	2	92 ^b^ (5352) ^b^	ND	ND	ND	ND	(50)ᵇ	ND	(50)ᵇ	ND
S0787	−	+	−	+	0	ND	ND	ND	ND	ND	ND	ND	ND	ND
S0821	+	+	+	+	6	ND	ND	ND	57 ^b^ (6284) ^b^	ND	ND	ND	ND	ND
S0843 ^a^	+	−	+	+	2	317 ^b^ (11974) ^b^	ND	ND	ND	ND	(94)ᵇ	ND	(94)ᵇ	ND
S0845	+	−	−	−	1	83 ^b^ (5009) ^b^	ND	ND	ND	ND	ND	ND	ND	ND
S1073 ^a^	−	+	−	+	0	ND	ND	ND	ND	ND	ND	ND	ND	ND
S1089	−	+	−	+	4	ND	ND	ND	ND	(125322) ^b^	310 ^b^ (2279) ^b^	(119) ^b^	310 ^b^ (2279) ^b^	(119) ^b^
S1106	+	+	−	+	4	ND	ND	ND	ND	ND	ND	140 ^c^ (2050) ^b^	ND	140 ^c^ (2050) ^b^
S1110	+	+	−	+	2	ND	ND	ND	ND	ND	ND	ND	ND	ND
S1137	+	+	−	+	2	ND	ND	ND	ND	ND	ND	210 ^c^ (3154) ^b^	ND	210 ^c^ (3154) ^b^
S1162	−	+	−	+	8	ND	ND	ND	ND	ND	ND	ND	ND	ND
S1192	−	+	−	+	1	ND	ND	ND	ND	ND	ND	ND	ND	ND
S1194	−	+	−	−	2	ND	ND	ND	ND	ND	61 ^b^ (491) ^b^	ND	61 ^b^ (491) ^b^	ND
S1196	−	+	−	+	2	ND	ND	ND	ND	ND	163 ^b^ (1425) ^b^	ND	163 ^b^ (1425) ^b^	ND
S1728	−	+	−	+	4	ND	ND	ND	ND	ND	ND	61 ^c^ (617) ^b^	ND	61 ^c^ (617) ^b^
S1729	−	+	+	+	7	ND	ND	ND	53 ^b^ (5170) ^b^	ND	ND	268 ^c^ (2943) ^b^	ND	268 ^c^ (2943) ^b^
S1730	−	+	−	+	5	ND	ND	ND	ND	ND	ND	142 ^c^ (2643) ^b^	ND	142 ^c^ (2643) ^b^
S1732	−	+	−	+	5	ND	ND	ND	ND	ND	ND	201 ᶜ (2201) ᵇ	ND	201 ^c^ (2201) ^b^
S2605	+	−	−	+	1	ND	ND	ND	ND	ND	ND	33 ^c^ (369) ^b^	ND	33 ^c^ (369) ^b^
S2606 ^a^	+	−	+	+	6	ND	(773) ^c^	806 ^b^	ND	ND	(90)^b^	961 ^b^ (6248) ^b^	(90) ^b^	961 ^b^ (6248) ^b^
S2719	+	−	−	+	2	77 ^b^ (4578) ^c^	(54) ^c^	ND	ND	ND	ND	ND	ND	ND
S2757	+	+	+	+	3	ND	(69) ^c^	ND	ND	ND	ND	364 ^c^ (5464) ^b^	ND	364 ^c^ (5464) ^b^
S3857	+	+	+	+	2	ND	52 ^c^ (5321) ^b^	ND	(768) ^b^	ND	ND	ND	ND	ND
S4497 ^a^	+	−	+	+	3	19 ^b^ (917) ^b^	286 ^b^ (12817) ^b^	631 ^b^	ND	ND	ND	ND	ND	ND
S4634	+	−	−	+	1	ND	ND	ND	ND	ND	ND	(148) ^b^	ND	(148) ^b^
S4738 ^a^	−	+	−	+	1	ND	ND	ND	ND	ND	219 ^b^ (2638) ^b^	ND	219 ^b^ (2638) ^b^	ND
90-11-287	−	+	+	+	3	ND	ND	ND	ND	ND	ND	192 ^c^ (2162) ^b^	ND	192 ^c^ (2162) ^b^
**No of occurrences/AHL**						5	7	3	3	1	11	17	11	17

With:

**Table 3:** AHLs in 32 Vibrionaceae strains tested against *C. violaceum* (*Cv*) and *A. tumefaciens* (*At*) using biomass or acidified EtOAc extracts and AHLs detected by UHPLC-DAD-QTOFMS; numbers demonstrate the peak area of the AHL in the chromatogram of the first run; numbers in brackets demonstrate the peak area of the AHL in the chromatogram of the second run; the total no. of occurrences/AHL does not include the reference strain *V. anguillarum* 90-11-287.

Strain	AHL Response in				No of AHL/Strain	Peak Area (1000 Counts) of AHL in Chromatogram								
	Biomass Screen		Extract Well Assay											
	*Cv*	*At*	*Cv*	*At*		C4	C6	C7	C8	C12	OH-C4	OH-C6	OH-C7	OH-C8
S0188 ^a^	−	+	+	+	9	ND	(114) ᶜ	178 ᵇ	ND	ND	32 ^b^ (161) ^b^	880 ^b^ (4822) ^b^	15 ^c^	176 ᵇ (1287) ᵇ
S0202	−	+	−	+	5	ND	ND	ND	ND	ND	ND	118 ^c^ (1659) ^b^	ND	ND
S0203 ^a^	−	+	+	+	7	ND	ND	ND	ND	ND	40 ^b^ (3709) ^b^	964 ^b^ (6479) ^b^	18 ^b^	193 ᵇ (1061) ᵇ
S0207	+	+	−	+	4	ND	ND	ND	ND	ND	ND	167 ^c^ (1807) ^b^	ND	ND
S0209 a	+	+	+	+	8	ND	(116) ᵇ	ND	ND	ND	17 c (342) b	627 b (4297) b	21c	106 ᵇ (1747) ᵇ
S0273 ^a^	+	+	+	+	7	ND	ND	ND	ND	ND	28 ^b^ (273) ^b^	896 ^b^ (5918) ^b^	13^c^	199 ᵇ (1007) ᵇ
S0344	+	−	−	−	2	92 ^b^ (5352) ^b^	ND	ND	ND	ND	(50)ᵇ	ND	ND	ND
S0787	−	+	−	+	0	ND	ND	ND	ND	ND	ND	ND	ND	ND
S0821	+	+	+	+	6	ND	ND	ND	57 ^b^ (6284) ^b^	ND	ND	ND	ND	ND
S0843 ^a^	+	−	+	+	2	317 ^b^ (11974) ^b^	ND	ND	ND	ND	(94)ᵇ	ND	ND	ND
S0845	+	−	−	−	1	83 ^b^ (5009) ^b^	ND	ND	ND	ND	ND	ND	ND	ND
S1073 ^a^	−	+	−	+	0	ND	ND	ND	ND	ND	ND	ND	ND	ND
S1089	−	+	−	+	4	ND	ND	ND	ND	(125322) ^b^	310 ^b^ (2279) ^b^	(119) ^b^	ND	ND
S1106	+	+	−	+	4	ND	ND	ND	ND	ND	ND	140 ^c^ (2050) ^b^	ND	ND
S1110	+	+	−	+	2	ND	ND	ND	ND	ND	ND	ND	ND	ND
S1137	+	+	−	+	2	ND	ND	ND	ND	ND	ND	210 ^c^ (3154) ^b^	ND	14 ᶜ (154) ᵇ
S1162	−	+	−	+	8	ND	ND	ND	ND	ND	ND	ND	ND	ND
S1192	−	+	−	+	1	ND	ND	ND	ND	ND	ND	ND	ND	ND
S1194	−	+	−	−	2	ND	ND	ND	ND	ND	61 ^b^ (491) ^b^	ND	ND	ND
S1196	−	+	−	+	2	ND	ND	ND	ND	ND	163 ^b^ (1425) ^b^	ND	ND	ND
S1728	−	+	−	+	4	ND	ND	ND	ND	ND	ND	61 ^c^ (617) ^b^	ND	ND
S1729	−	+	+	+	7	ND	ND	ND	53 ^b^ (5170) ^b^	ND	ND	268 ^c^ (2943) ^b^	ND	ND
S1730	−	+	−	+	5	ND	ND	ND	ND	ND	ND	142 ^c^ (2643) ^b^	ND	ND
S1732	−	+	−	+	5	ND	ND	ND	ND	ND	ND	201 ᶜ (2201) ᵇ	ND	ND
S2605	+	−	−	+	1	ND	ND	ND	ND	ND	ND	33 ^c^ (369) ^b^	ND	ND
S2606 ^a^	+	−	+	+	6	ND	(773) ^c^	806 ^b^	ND	ND	(90)^b^	961 ^b^ (6248) ^b^	57 ^b^ (1663) ᵇ	38 ᵇ (285) ᵇ
S2719	+	−	−	+	2	77 ^b^ (4578) ^c^	(54) ^c^	ND	ND	ND	ND	ND	ND	ND
S2757	+	+	+	+	3	ND	(69) ^c^	ND	ND	ND	ND	364 ^c^ (5464) ^b^	ND	ND
S3857	+	+	+	+	2	ND	52 ^c^ (5321) ^b^	ND	(768) ^b^	ND	ND	ND	ND	ND
S4497 ^a^	+	−	+	+	3	19 ^b^ (917) ^b^	286 ^b^ (12817) ^b^	631 ^b^	ND	ND	ND	ND	ND	ND
S4634	+	−	−	+	1	ND	ND	ND	ND	ND	ND	(148) ^b^	ND	ND
S4738 ^a^	−	+	−	+	1	ND	ND	ND	ND	ND	219 ^b^ (2638) ^b^	ND	ND	ND
90-11-287	−	+	+	+	3	ND	ND	ND	ND	ND	ND	192 ^c^ (2162) ^b^	ND	ND
**No of occurrences/AHL**						5	7	3	3	1	11	17	5	6

The authors would like to apologize for any inconvenience caused to the readers by these changes.
