# Supplementary material for: Global and Phylogenetic Distribution of Quorum Sensing Signals, Acyl Homoserine Lactones, in the Family of Vibrionaceae
Source: Mar Drugs. 2014 Nov 20;12(11):5527–46. doi: 10.3390/md12115527 (PMC4245543; doi:10.3390/md12115527)
Supplement: Supplementary File 1 [file marinedrugs-12-05527-s001.pdf]

Supplementary Information

Table S1. Screening for general AHL response using AHL standards in well assay against *C. violaceum* (*Cv*) and *A. tumefaciens* (*At*) with three 10-fold dilutions; +++ strong, ++ medium, + weak, − no response. HSL = homoserine lactone.

**Name**		**Concentration of Dilutions in μmol L^{-1} for Cv Assay**			**Concentration of Dilutions in μmol L^{-1} for At Assay**			**AHL Response in Extract Well Assay**					
								Cv	***Cv***	***Cv***	***At***	***At***	***At***
	**FW**	**1**	**1:10**	**1:100**	**1**	**1:10**	**1:100**	**1**	**1:10**	**1:100**	**1**	**1:10**	**1:100**
C4-HSL; *N*-Butyryl-DL-HSL	171	4089	409	41	4089	409	41	+++	++	−	−	−	−
C6-HSL; *N*-Hexanoyl-DL-HSL	199	50	5.0	0.50	5019	502	50	+++	++	−	+++	++	−
C8-HSL; *N*-Octanoyl-DL-HSL	227	44	4.4	0.44	4.4	0.44	0.044	++	+	−	+++	+++	−
C10-HSL; *N*-Decanoyl-DL-HSL	255	3133	313	31	39	3.9	0.39	+	+	−	+++	+++	−
C12-HSL; *N*-Dodecanoyl-DL-HSL	283	3176	318	32	353	35	4	+	−	−	++	++	−
C14-HSL; *N*-Tetradecanoyl-DL-HSL	311	3211	321	32	3211	321	32	−	−	−	++	++	++
C18-HSL; *N*-Octadecanoyl-L-HSL; oDHL	367	2721	272	27	2721	272	27	−	−	−	++	−	−
3-OH-C4-HSL; *N*-(3-Hydroxybutyryl)-L-HSL; HBHL	187	5342	534	53	5342	534	53	+	−	−	++	++	−
OH-C6-HSL; *N*-(3-hydroxy-hexanoyl)-L-HSL	215	4.6	0.46	0.05	46	4.6	0.46	+	−	−	++	++	−
OH-C12-HSL; *N*-(3-hydroxy-dodecanoyl)-L-HSL	299	3340	334	33	3.3	0.33	0.033	++	+	−	++	++	−
O-C4-HSL; *N*-(3-Oxobutyryl)-L-HSL; OBHL, 3-oxo-C4-HSL	185	5400	540	54	5400	540	54	+++	+	−	++	++	−
O-C6-HSL; *N*-(beta-Ketocaproyl)-L-HSL	213	47	4.7	0.47	4.7	0.47	0.047	+++	+	−	++	++	−
O-C8-HSL; *N*-(3-oxo-Octanoyl-L)-HSL	241	41	4.1	0.41	4.1	0.41	0.041	+++	+	−	+++	+++	+++
O-C10-HSL; *N*-(3-oxo-decanoyl-L)-HSL	269	371	37	3.7	3.7	0.37	0.037	+	+	−	+++	+++	++
O-C12-HSL; *N*-(3-oxo-dodecanoyl-L)-HSL	297	336	34	3.4	3.4	0.34	0.034	−	−	−	+++	+++	++
6-Ph-3-oxo-C6-HSL; *N*-(6-Phenyl-3-oxohexanoyl)-L-HSL; PhOHHL	289	346	35	3.5	3.5	0.35	0.035	+	−	−	+++	+++	+++

Figure S1. World map with sampling sites of the AHL-positive Vibrionaceae strains and approximate climate zones. Geographic data is available in Table 1. Map data ©2014 Google.

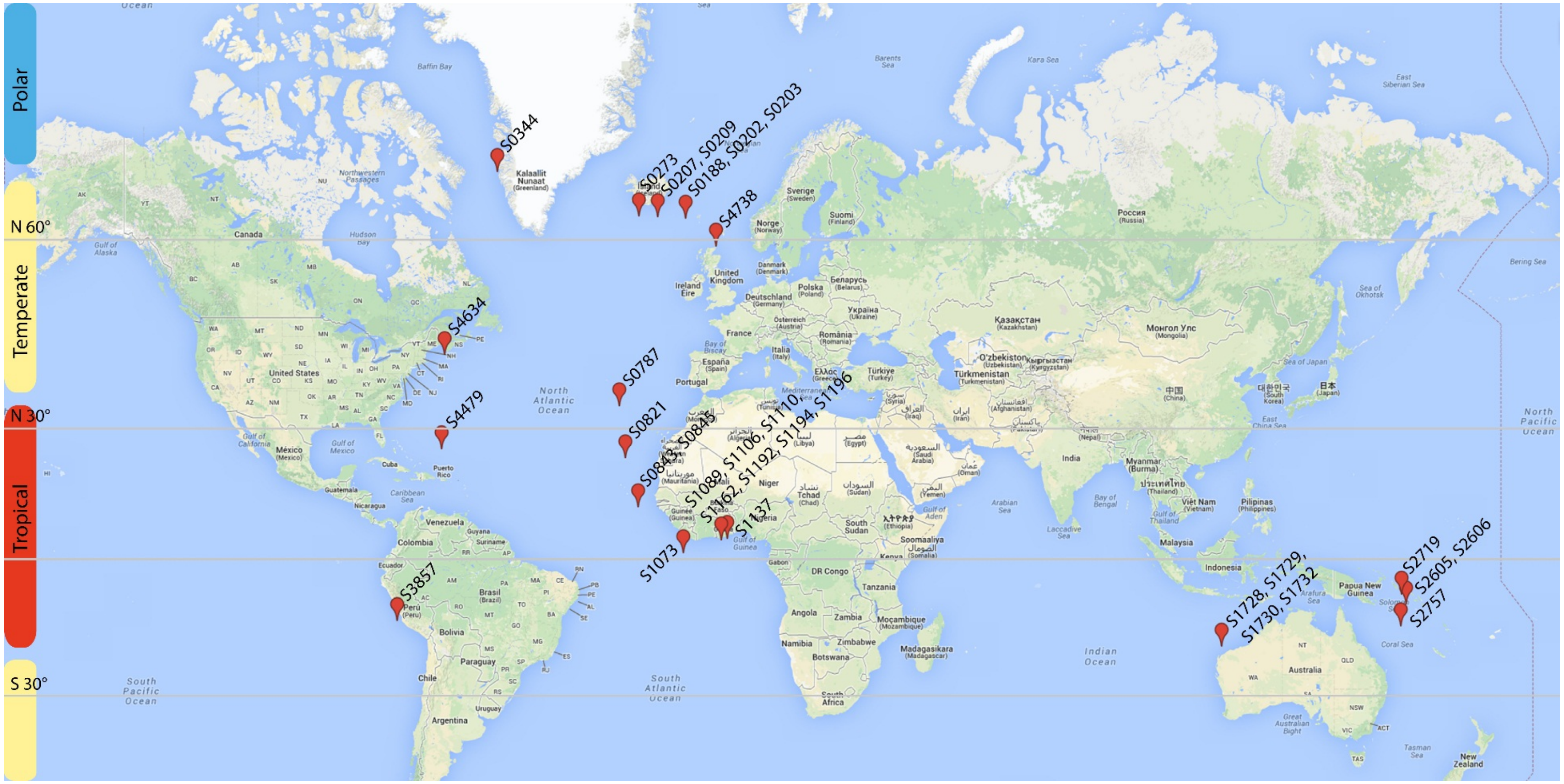

© 2014 by the authors; licensee MDPI, Basel, Switzerland. This article is an open access article distributed under the terms and conditions of the Creative Commons Attribution license (http://creativecommons.org/licenses/by/4.0/).
